# Social Psychology of and for World-Making

**DOI:** 10.1177/10888683221145756

**Published:** 2023-01-11

**Authors:** Séamus A. Power, Tania Zittoun, Sanne Akkerman, Brady Wagoner, Martina Cabra, Flora Cornish, Hana Hawlina, Brett Heasman, Kesi Mahendran, Charis Psaltis, Antti Rajala, Angela Veale, Alex Gillespie

**Affiliations:** 1 University of Copenhagen, Denmark; 2 Université de Neuchâtel, Switzerland; 3 Utrecht University, The Netherlands; 4 Aalborg University, Denmark; 5 Oslo New University College, Norway; 6 London School of Economics and Political Science, UK; 7 York St. John University, UK; 8 The Open University, Milton Keynes, Buckinghamshire, UK; 9 University of Cyprus, Nicosia, Cyprus; 10 University of Eastern Finland, Kuopio, Finland; 11 University of Oulu, Pohjois-Pohjanmaa, Finland; 12 University College Cork, Ireland

**Keywords:** cultural psychology, ethics, imagination, methodological pluralism, social psychology, world-making

## Abstract

**Academic Abstract:**

Social psychology’s disconnect from the vital and urgent questions of people’s lived experiences reveals limitations in the current paradigm. We draw on a related perspective in social psychology^1^—the sociocultural approach—and argue how this perspective can be elaborated to consider not only social psychology as a historical science but also social psychology of and for world-making. This conceptualization can make sense of key theoretical and methodological challenges faced by contemporary social psychology. As such, we describe the ontology, epistemology, ethics, and methods of social psychology of and for world-making. We illustrate our framework with concrete examples from social psychology. We argue that reconceptualizing social psychology in terms of world-making can make it more humble yet also more relevant, reconnecting it with the pressing issues of our time.

**Public Abstract:**

We propose that social psychology should focus on “world-making” in two senses. First, people are future-oriented and often are guided more by what could be than what is. Second, social psychology can contribute to this future orientation by supporting people’s world-making and also critically reflecting on the role of social psychological research in world-making. We unpack the philosophical assumptions, methodological procedures, and ethical considerations that underpin a social psychology of and for world-making. Social psychological research, whether it is intended or not, contributes to the societies and cultures in which we live, and thus it cannot be a passive bystander of world-making. By embracing social psychology of and for world-making and facing up to the contemporary societal challenges upon which our collective future depends will make social psychology more humble but also more relevant.

History reveals that our social world is ever-changing, often rhyming, but never repeating. The world of tomorrow is forged, sometimes unpredictably, in contestation over visions of the future. While there are undoubtedly stable patterns across time and cultures that should be understood, such as evolutionary mechanisms, basic cognitive processes, and personality traits, we argue that social psychology can become even more useful and relevant by also researching change and possibility. Social psychology needs to go beyond what *is*, to research what *could be*, while not shirking any responsibility for what *becomes*. Yet, the prevailing assumptions and methods of social psychology are best suited to conceptualizing a static world. Over-reliance on experimentation in a vacuum is like using a camera to take a snapshot of human life that is in motion. Although an individual photograph, or series of photographs, can build an image of human thought, feeling, or action, it fails to engage with the purposes guiding human life; what people are trying to achieve. This over-reliance on experiments and positivist assumptions creates a disconnect between psychological conceptualizations of phenomena and their historical and future manifestation and thus also between the aspirations of our discipline and its actual impact.

We introduce the idea of social psychology *of* and *for* world-making. World-making refers to the fact that humans at both an individual and collective level contribute to the making of societies, social relations, and cognition (i.e., memory aids, distributed cognition). We distinguish between the study of people making worlds and the role of social psychology in contributing to this world-making. Social psychology has the theories, ethics, and methods to study people as world-makers, with the resultant knowledge not only describing the world but also contributing to the transformation of societies, social relations, and cognition. We argue that: (a) ontologically, the world is always changing; (b) epistemologically, social psychologists are not observers separated from the world, but rather they use interventions and imagination to learn about the world by participating in it; (c) ethically, researchers have a responsibility to critically consider how people, in general, and social psychologists in particular, are involved in world-making; and (d) methodologically, social psychology needs methodological pluralism to engage with dynamic social phenomena. We conclude by demonstrating how social psychology of and for world-making can address current limitations of the discipline and make it more relevant for the future.

## Prologue: The Two Current Crises of Social Psychological Science

Our contribution is motivated in part by the so-called replication crisis and the dominant responses to it. The replication crisis was instigated by the finding that only one third of the experimental studies published in premier psychology journals could be replicated (Open Science Collaboration, 2015). In a later attempt to replicate another cohort of studies published in *Nature* and *Science* between 2010 and 2015, 62% of the findings were replicated (Camerer et al., 2018). The fallout of these high-profile collaborative efforts has been met with several within-paradigm attempts to advance social psychological science (Zwaan et al., 2018), for example, by improving survey and experimental methods by preregistering hypotheses, developing more stringent research designs, advancing sampling procedures, sharing data, and undertaking more sophisticated statistical analyses (Chambers, 2017; Power & Velez, 2020).

Although we welcome efforts to develop a more robust and credible social psychology, the ontological, epistemological, ethical, and methodological assumptions underlying the current desire to replicate findings should also be examined. This crisis highlights the prominence of decontextualized and ahistorical examination of social psychological phenomena (Akkerman et al., 2021; Baucal et al., 2020; Gergen, 1973; Sullivan, 2020). We do not intend to offer a solution to the “replication crisis.” Rather, our view is that this decontextualization of dynamic processes and the separation of psychological processes from historical context creates an overly narrow social psychological paradigm—in terms of theory and methods—which undermines not only scientific validity but also societal relevance. As such, our argument is that the “replication crisis” offers a starting point to think about recontextualizing human behavior, which necessitates a broadening of the paradigm. This conclusion is further strengthened by considering social psychology’s second crisis.

The second crisis is the Western, Educated, Industrial, Rich, and Democratic (WEIRD) problem, in which “universal” theories of human thought, feeling, and behavior are generated through over-reliance on samples that represent atypical psychological functioning. The psychological uniqueness of WEIRD populations suggests that sociocultural processes, steeped in deep time, lie at the basis of variation in psychological phenomena (Henrich, 2020; Henrich et al., 2010; Schulz et al., 2019). The problem is that generating psychological knowledge from an atypical, nonrepresentative sample of humanity, directly or indirectly, becomes part of both understanding and forming the world. The over-reliance on WEIRD samples leads not only to limited psychological insight but also to bad world-making. Bad world-making, in contrast to good world-making, is the process by which systemically distorted, and therefore inaccurate research generates knowledge that informs everyday understandings of phenomena, economic decisions, and political, educational, and legal policies that are self-interested, incomplete, and do not feed forward into just and empowering world-making. The WEIRD problem, like the replication crisis, highlights a further limitation of the current social psychological paradigm and, therefore, offers a second motivation to make this paradigm more responsive to, reflective of, and reflexive about human behavior.

In this article, we argue for a reexpansion of the social psychological paradigm and methods, aligned with historical and contemporary perspectives calling for a contextually engaged, historically situated, methodologically plural, and culturally embedded social psychology (Asch, 1952/1987; Bruner, 1990; Cole, 1996; Markus & Kitayama, 1991; Power & Velez, 2020; Power et al., 2018; Rozin, 2001; Shweder, 1991, 2003; Sullivan, 2020). Our contribution is to consider social psychology not only as a historically embedded discipline but also as one that is future-oriented. While previous meta-theoretical arguments have situated “social psychology as history” (Gergen, 1973, 2015; Jahoda et al., 1932/1971), we put the emphasis on “social psychology as future.” Although social psychology predominantly uses scientific methods to test predictions from theoretically derived hypotheses, these theories are reflections of contemporary culture. From this perspective, social psychological theories may be considered either tool capable of summarizing phenomena or torches illuminating fundamental causal forces uniting seemingly disparate phenomena (Sullivan, 2020; also see Stam, 2010). This positioning aims to recontextualize social psychology within a critical-historical framework, which is a political agentic agenda manifesting from the sociocultural traditions in psychology.

We seek to advance this sociocultural recontextualization of social psychology. We argue that humans are not only embedded in the past; they also live, at a psychological level, in many potential futures (Boesch, 1991; Glăveanu, 2020). This expansion takes us beyond the traditional historical–sociocultural paradigm and invites us to articulate the conceptualization of social psychology as world-making in two ways. First, we illustrate how social psychology can study people as world-makers. Second, we illuminate how social psychologists themselves contribute to this world-making. Accordingly, we call for a deeper reflection on human behavior as not only historical but also fundamentally future-oriented (Akkerman et al., 2021; Wagoner, 2017; Wagoner et al., 2017; Zittoun & Gillespie, 2015, 2018, 2020).

One can characterize much social psychological research as a search for ahistorical laws about how the past *pushes* people into the future. However, we advocate for research on how imaginings of the future *pull* humans forward. Such a paradigm shift requires us to reconsider social psychology’s ontological, epistemological, methodological, and ethical assumptions.

Many of the authors of this article do not identify (solely) as social psychologists. Some are cultural, sociocultural, critical, developmental, political, or educational psychologists (or some combination thereof). Each author has a range of varying opinions, perspectives, and orientations regarding their understanding of, and research on, contextually embedded people. Each author agrees that a conceptualization of “social psychology of and for world-making” makes a significant contribution to developing psychological science more broadly. At the time of publication, the 13 authors of this manuscript range from PhD student to professor, have a range of nationalities and expertise, and work in many different countries. But, despite this breadth of authorship, the present article is inescapably a product of the authors. The constraints of generalizability relate to our intellectual heritage (largely sociocultural psychology, and also informed by the historical tradition of American pragmatism), our geographic location (we are all located in European universities), and we are well-to-do academics, which shapes our shared assumptions about, and aspirations for, a social psychology that not only theorizes humans but also contributes to and enriches humanity. This approach might seem naive to people in harsher circumstances.

We introduce four propositions for examining dynamic social psychological phenomena from a sociocultural psychological perspective. Specifically, we argue: (a) ontologically, the world is not like a clock comprising predefined cogs that have been set in motion, rather, it is a genuinely developmental world in which new species, capabilities, comprehensions, technologies, and events are being both created and lost; (b) epistemologically, social psychologists are not observers who are aloof from the world, rather, they use interventions and their imagination to learn about the world by participating in it; (c) ethically, social psychologists are embedded in and contributing to the development of the social world and thus have ethical responsibilities not only in conducting research but also in producing knowledge and interventions; and (d) methodologically, social psychology needs methodological pluralism capable of comprehending the qualitative and future-oriented nature of social phenomena as they develop. In what follows, we elaborate on each of these propositions, based on diverse and interdisciplinary sources and authors in social psychology and beyond and conclude by returning to the implications of these propositions for social psychology.

## Ontology for a Social Psychology of and for World-Making

Ontologies conceptualize the composition of the world being investigated and focus attention on specific phenomena. Arguably, the most fundamental distinction is between ontologies that give priority to either things or processes (Brinkmann, 2013; Brown et al., 2011; Markova, 1982; Shweder, 1996; Stenner, 2017). The key issue is whether the elements of the world are given a priori, and research is the mere documentation of how these elements move about or whether the world is genuinely creative with novel phenomena emerging. An example of an ontology of things is any theory that assumes invariant variables that have determined relationships, such as the metaphor of the mind as computer, where the hardware has fixed functions in fixed relationships that are unchanged by the software (Leary, 1990). An example of a process ontology is developmental psychology, where the focus is on how cognitive processes, personality traits, and psychological tendencies develop and change over the life course through experience within social and cultural relationships (Valsiner, 2007a, 2007b; Zittoun & Gillespie, 2020). Thus, within a process ontology, the role of research is to understand how new phenomena that did not exist a priori come into being. While an ontology of things is powerful for understanding the elements that make up the universe, it is not so useful for understanding how humans will respond to collective challenges (e.g., pandemics, climate change)—where new responses may be invented and humans are responsible for responding and thus altering the course of events. Nor is an ontology of things particularly useful for understanding how humans are agents in creating their own collective future because we need to be able to conceptualize how new goals, institutions, and practical technologies that we cannot yet imagine will come into existence.

The social psychology of and for world-making requires a process ontology that focuses attention on the creative emergence of new phenomena. The issue is not merely that elements may recombine into new configurations but that new phenomena may genuinely emerge (e.g., new mnemonic techniques, cognitive heuristics, communication platforms, and collective forms of organizing; Dewey, 1910; Vygotsky, 1931/1994). This paradigmatically distinct ontological position, despite being rare, has an established history—from the ancient philosophy of Heraclitus (“no man ever steps in the same river twice”), through the Renaissance (de Spinoza, 1677), to more contemporary philosophy (Barad, 2007; Bergson, 1938; Rorty, 1981; R. M. Unger, 2007; Whitehead, 1979; for a review see Rescher, 1996). Moreover, there is a close affinity between psychology and process philosophy, as is evident in the writings of John Dewey and William James, both of whom made important contributions to process philosophy and helped found psychology as a discipline (James, 1890; Mead, 1932; Stenner & Brown, 2009; Zavershneva & van der Veer, 2018). Process ontologies are essentially an expansion of Darwin’s theory of evolution into a philosophical position (Dewey, 1910; Gillespie, 2006). Before Darwin, “species” were conceptualized as immutable elements, as pre-given cogs in the clockwork universe. By demonstrating how species evolve, Darwin challenged the sacred assumption of predefined ontological types, revealing that even the most sacred of things (i.e., humans) were in development and unfinished. Process ontologies also share many assumptions with developmental sciences, which also postulate the primacy of dynamics, movement, and change (Bornstein & Lamb, 2015; Valsiner et al., 2009; van Geert, 2019; Wagoner et al., 2014; Zittoun & Gillespie, 2020) and with broader theoretical work on all life developing in the direction of surprise reduction (Friston, 2010). For us, in contrast to much contemporary social psychological research that has a “static” (not process) ontology as its philosophical basis, a developmental or processual stance is necessary for studying humans changing within a changing world. This implies that we consider the open-ended nature of life and societies, which we are becoming within a social world that is in the making.

There are many sub-traditions within psychology that have a more or less explicit process ontology, including genetic social psychology (Psaltis, 2015; van Geert, 2019; Witherington & Boom, 2019), ecological psychology (Bronfenbrenner, 1986; J. J. Gibson, 2014), feminist psychology (R. K. Unger & Crawford, 1992), critical psychology (Prilleltensky & Nelson, 2017), societal psychology (Himmelweit & Gaskell, 1990), community psychology (Orford, 1992), liberation psychology (Martín-Baró, 1994), narrative psychology (Schiff, 2017), and field social psychology (Power & Velez, 2022). The process ontology that we focus on is dialogism (Gillespie & Cornish, 2010; Marková, 2016) because it has an explicit process metaphor, namely, “dialogue.”

Dialogism has a long history in European philosophy, literature, and psychology (Bakhtin, 1983; Linell, 2009; Marková, 2016). Dialogism focuses on concrete relations—between turns of talk, between self and other, and between the person and the environment. Dialogism as a paradigm is not limited to talk; it also includes relational theorizing of practical activity, semiotic processes, cognitive processes (e.g., internal dialogues), and affective processes. In all cases, it is the relation between socially situated humans that is primary (Marková, 2016; Mead, 1932); all human action and interaction is conceptualized as part of a dialogue, with each turn in the dialogue being simultaneously a response to what went before and also, crucially for our present concern, an orientation to and attempt to shape the future (Linell, 2009).

Dialogism, as an ontology, focuses on relations and interactions between people and the social and material world, with each turn in the interaction responding to what went before and contributing to what will come. It foregrounds creative emergence, subjective experience, and ethical responsibility for one’s role in cocreating the future. Accordingly, we argue, it is a well-suited ontology from which to develop an epistemology to study the social psychology of and for world-making.

## Epistemology for a Social Psychology of and for World-Making

Epistemology is the study of knowledge, particularly how it is produced and justified. Traditionally, epistemology has been used to distinguish truth from falsity and thus justify academic knowledge. In this mode, it examines the basis for believing in a given theory: Is the evidence based on experience, experiments, or rational thought? However, epistemology is not only an issue for scientists. People talking, reading the news, and debating online are also creating knowledge (i.e., common sense). These beliefs and representations are created through social interaction; regardless of their veracity, they have consequences through guiding people’s actions. Thus, a social psychology of world-making needs an epistemology that is applicable both to scientific knowledge and to the creation of common sense in everyday life (Duveen, 2008; Marková, 2008, 2016).

Arguably, in both science and common sense, the minimum unit for knowledge production is the self–other–object triadic relation (Marková, 2003; Moscovici, 2008; Zittoun et al., 2007). This triadic epistemological model depicts knowledge as a function of both practical action (relating to the object) and communication (relating to the other). A crucial insight of sociocultural psychology is that altering the dynamics of the self–other–object relation, skewing the generative heart of knowledge production, alters the knowledge produced (Jovchelovitch, 2007). For example, children develop knowledge differently depending on whether they are in symmetrical or asymmetrical self–other relations; novel forms of knowledge are more likely to emerge in symmetrical rather than asymmetrical social relations (Psaltis & Duveen, 2006). Equally, in the public sphere (i.e., forums for public debate), where issues of common concern are debated and democracies evaluate their governments, distortions of self–other relations can also skew the knowledge produced (Habermas, 1970). For instance, excluding stakeholders, giving undue regard for status, or denying people the legitimacy to either speak or be heard are common manipulations in the epistemology of creating common sense, public opinion, and widespread beliefs (Fricker, 2007; Mahendran, 2018). Precisely because humans make the future through the public sphere, it is a contested and often subverted space where vested interests attempt to guide the course of knowledge production, potentially leading to systematically distorted outcomes (Habermas, 1970; Sloan, 1996). Moving to the epistemology of science, limiting distortions of the self–other–object relation through clearly documented procedures and shared data has been central to the success of science (Ziman, 1991). Peer review aims to curtail the impact of status. Anyone can submit a manuscript for publication and, ideally, it will be evaluated based purely on its substance.

One key epistemological issue for scientific knowledge is generalization. Traditionally, this is conceptualized in terms of creating a general theory based on specific observations. In social psychology, it is usually based on aggregating statistical results and observing repeated measures that are deemed to predict future occurrences. The “general theory” is conceptualized outside of time (i.e., assuming an ontology of things); at best, it is a question of applying insights to other geographical contexts. A future-oriented social psychology reframes generalization as a question of applying knowledge from a partially known past to a largely unknown future (Cornish, 2020a, 2020b; Marková et al., 2020). The future will never replicate the past; yet, the past is our only guide to the future. A future-oriented epistemology accepts the limitation of all knowledge in the face of the future (and adapts). It acknowledges that one-off particulars can have cascading consequences that end up changing the course of history (Goldstone, 1998). Instead of seeking mechanical laws of history, future-oriented social psychology poses a fundamental question: Are we trying to predict people or liberate them from prediction? Are we trying to discover the limits of human nature or identify new potentials in human nature?

It is thus necessary to look beyond the traditional focus of how knowledge is developed toward the more pragmatist question of what the knowledge will be used for (Addams, 1910; James, 1907). Knowledge in everyday life, such as common sense and social representations, enables people to interact with social phenomena (e.g., genetically modified food, pandemics and contagion, and out-groups) and make those phenomena communicable in social relations (Bauer & Gaskell, 2008). In science, knowledge does not merely describe or “mirror” (Gergen, 2015) the world; it has effects. The various ways in which science has conceptualized mental illness, for example, have led to exclusion, confinement, therapy, or medication (Foucault, 1973). This is a paradigmatic shift—away from viewing knowledge as a mere description of the world toward recognizing it as constitutive in world-making.

Habermas (1968) argued that, as a causal force in the world, knowledge can serve three broad interests. First, it can be used for technocratic control. For example, it was argued that behaviorism should aim to predict and control behavior (Danziger, 1990; Watson, 1913). Second, knowledge can be used to understand action. Studies of historical texts, observations of human behavior, and first-person accounts of participants provide a hermeneutic understanding of human action from the inside (motives, reasons). Third, it can be used for emancipation, liberating people from constraints. For example, Freud liberated (to some extent) people from the constraints of the unconscious (Zimbardo, 2012). Social psychology has served all three interests. It has informed research on predicting and controlling human behavior (e.g., advertising, opinion polling), interpreting human experience (e.g., phenomenology and selfhood), and empowering human activity (e.g., participant action research and intervention research). However, these interests do not receive equal attention: the search for funding skews research toward technocratic questions in the service of vested interests, advancing the priorities of governments, philanthropic donors, and corporations.

One way to conceptualize the feed-forward impact of knowledge (lay or scientific) in world-making is in terms of “looping effects,” whereby the knowledge produced by social scientists alters the phenomena they purport to describe (Hacking, 1995, 2007; N. Haslam, 2016; Richards, 2002). There is a people–scientists–people epistemological self–other–object triangle: the social scientists take people as their object, producing knowledge about people; however, this knowledge is also created in dialogue with people and, moreover, becomes part of the people, changing who they are and what they can do. Social science questions arise out of common sense and generate ideas and theories that feed back into common sense, thus altering the phenomena being studied. In this sense, humans are now post-Freudian, post-behaviorist, and post-Milgram, and increasingly post-cognitive, post-priming, and post-nudging. Humans are “post” these psychological paradigms because the paradigms have spread into common sense: People query their unconscious, use behaviorism on their children, keep their biases in check when making investments, and realize when they are being nudged.

Indeed, much social science has focused on describing people as they are, and then these descriptions unintentionally loop back into common sense. However, a sociocultural epistemology of world-making takes the idea of looping effects one step further, arguing that genuinely emancipatory psychological knowledge generates possibilities for how people could be. Given the power of these dynamic looping effects, the question changes from who we “really” are to who we could become. Humans are simultaneously living in the present and striving toward a future that is motivating precisely because it differs from the present (Akkerman et al., 2021). Imagination is not secondary to the sociocultural world; rather, it is the horizon of world-making (Zittoun & Gillespie, 2015, 2018, 2020) What are the potentialities of being human? How can the sociocultural-technological world, which we create, bring out these potentials (e.g., Psaltis, 2012)? And how can humans collectively participate in steering their own self-making (e.g., Mahendran et al., 2022)?

Attempting to support people in individually and collectively imagining and striving toward various possible futures comes with responsibilities. In the next section, we outline two ethical principles associated with our epistemological interpretations and based on our ontological principles of social psychology of and for world-making.

## Ethics for a Social Psychology of and for World-Making

The ethics of research is typically considered in terms of defined rules for collecting and storing data (e.g., informed consent, transparency, privacy). This widespread approach to ethics has a “static” rather than a “process” ontology. Emphasis is placed on the separation of researcher and participant. However, with a social psychology of and for world-making, a more fundamental layer of ethics appears: one that is attentive to participants and researchers as human agents, each acting and interacting with particular positions and perspectives in the relational, communicative, and consequential space of a research project (Akkerman et al., 2021). This is a reconceptualized and re-expanded ethical framework, based on a dialogical process ontology.

Focusing on world-making necessarily foregrounds responsibility (Marková, 2016). For example, although the dialogue is filled with familiar refrains and tropes, the form and outcome are always, to some extent, unpredictable—not given *a priori*; they are co-created dynamically through the dialogue. Insofar as the outcome is not given a priori, each participant in the dialogue has a responsibility for making the future. Even being passive is contributing (e.g., acquiescing). This responsibility for one’s role in world-making is important not only for individuals but also for disciplines such as social psychology. Accordingly, we need to focus on the divergent perspectives of the researcher and the researched, question how they come together to make knowledge, and reflect on what that knowledge is used for and who it benefits. Is it knowledge to enable participant action or advance an academic career?

First, researchers need to approach research participants with reflexivity and sensitivity. Both parties in the research have hopes and fears (e.g., desired findings, uncertainty about being evaluated), self-presentation (e.g., wanting to look competent, wanting to give the right answers), and creative insights (e.g., for interpreting the results, suggestions for improving the research). Thus, researchers need to relate to research participants as fellow human beings and use their common language and culture to better understand participants’ interests within the research and share information on the research, remaining open to feedback. The point is that pushing for distance and treating participants as objects can create a very unnatural context that makes generalization dubious. Instead, researchers need to approach participants as fellow humans who are engaged in their own projects of world-making (just like the researcher; Cornish, 2020a).

Second, researchers need to acknowledge and include participants’ perspectives alongside their own perspective in capturing how people individually and collectively think, feel, act, and produce the future. The perspective of a research participant can never be disregarded, as it is the primary perspective for revealing reality as it is actualized and made by and thus *for* them: What is perceived, experienced, valued, imagined, and projected in the future, regardless of whether any of this is observable to another. The researcher is, besides a professional, also a person with a particular private life, history, knowledge, and opinions about the world. The more unfamiliar the researcher is with the cultural, social, and life conditions of a participant, the more important it is for the researcher to explore these and understand the participants’ perspectives before making any evaluative claims (Akkerman et al., 2021); this step is often neglected (e.g., in psychological deficit models or educational qualifications like “the unmotivated students,” or black-box participants in online study pools). In sociocultural psychology, the value of the researcher’s perspective resides precisely in the attempt to systemically *render* persons’ experiences and acts in the fullness of their lives (Bakhtin, 1983; Geertz, 1973; Vygotsky, 1986), highlighting their logics and particularities across historical and geographical contexts. Put another way, the challenge for researchers lies less in predicting what participants will do and more in making sensible whatever it is that participants do.

Finally, research does not merely describe or mirror lives, events, and developments of the people being studied; research has consequences for which it is answerable. Research revoices and reproduces perspectives, always centering some definition of the situation over others (Akkerman et al., 2021), reifying these in scientific discourse as significant (Akkerman & Niessen, 2011; Packer & Goicoechea, 2000), and making societal claims of relevance, conclusions, and implications to be regarded by others (i.e., scientists, stakeholders, and participants). In this way, researchers co-opt participants into a project of world-making, which could have consequences unintended by the research and even undesired by the participants. Ethics concerns what Bakhtin referred to as *answerability*, meaning awareness of ones’ own consequential role in the account, and hence the need for responsibility and responsivity to participants and the public. Concrete acts—or “answerable deeds”—that are responsive to and address others and their voices constitute the axiological center of human practice, connecting actions in the moment-to-moment lived reality to large-scale historical developments of societies (Stetsenko, 2016).

## Methodology for a Social Psychology of and for World-Making

Researching future-oriented questions requires ethically responsible multi-methodological frameworks capable of conceptualizing the creation of as-yet-unknown psychological, social, cultural, and political phenomena over time (Power & Velez, 2020). In this section, we outline methodological approaches for social psychology of and for world-making, arguing for a reexpansion of the social psychological toolkit.

The methods highlighted are not an exhaustive list. Moreover, we are not merely advocating for the types of mixed methods or field social psychological research that have recently been articulated (Power & Velez, 2022; Power et al., 2018). Rather, we provide illustrations of the types of methods most congruent with social psychology of and for world-making. Other methods, or innovative combinations of methods, can help examine how human activities are directed to achieve future goals. Moreover, they also enable us to comprehend the processes unfolding where new phenomena emerge, which are not fully predictable. Consequently, a focus on contextualization, processes, and future orientation implies that replication of findings is not the main point of interest. The goal is to refine our tools for accessing and analyzing unpredictable and nonrepeatable events in the real world and for better comprehending human minds and mentalities across times and cultures. There will be some generalities that can be transferred between cases, but each will also have its specificities. Therefore, this methodological orientation departs from concerns with experimentation and replication that are paramount in the dominant social psychological paradigm. Instead, the focus is on methods that are consonant with a process ontology that guides considerations of ethical knowledge creation. Although these methods are not novel in and of themselves, the innovation of the forthcoming section is to reconceptualize these methods as social psychological methods of and for world-making. In doing so, we operationalize our ontological, epistemological, and ethical framework with illustrations from recent research.

### Methods for a Social Psychology *of* World-Making

World-making begins with an imagination of the future, a symbolic construction not of what is but of what could be, which then guides activity (Power & Velez, 2020; Vygotsky, 1931/1994; Wagoner et al., 2017; Zittoun & Cerchia, 2013; Zittoun & Gillespie, 2015, 2018, 2020). Imagination can be defined as a phenomenological break from the here-and-now of immediate and proximal stimuli, with the stimuli of experience becoming purely symbolic (i.e., thoughts, images, feelings; Zittoun & Gillespie, 2015). Despite being highly personal, the act of imagining is deeply cultural and social: Imagination builds on ideas and images circulating within a cultural environment and is shaped by the access to (and censorship of) cultural elements that can be used as symbolic resources for imagining (e.g., books and films). Imagining can also be socially endorsed (e.g., celebrated as visionary) or sanctioned (e.g., taboo). Therefore, imagination, being simultaneously personal and cultural, necessitates a broad methodological approach.

People engage, for example, in both micro (e.g., individual, cognitive) and macro (e.g., collective, political) acts of world-making. Individual acts of world-making include everyday planning, such as deciding when and where to socialize. Collective world-making includes activities such as agitating for social change around a shared vision for the future. Acts include voicing concerns, challenging plans, voting in a new government, or protesting against political injustice. This variety of contexts of world-making means that a variety of methods are required.

We outline three types of methods that transcend an opposition between qualitative and quantitative research paradigms to comprehend how individuals and collectives engage in the psychology of world-making. Case studies, surveys, and naturally occurring text data are presented briefly as separate methods. Despite being presented sequentially, in practice, a combination of methods can be used in conjunction to more holistically comprehend how people engage in world-making (Flick, 2011; Power et al., 2018). Overall, these three methods are examples of a broader array of methods that can legitimately be used in world-making.

#### Case Studies

Case studies are able to capture complex relationships and dynamics as they change through time, which, as we have argued above, is a defining characteristic of social and psychological phenomena (Marková et al., 2020). The following three principles are central to doing a proper case study:

First, the phenomenon of interest needs thorough contextualization. This may involve an analysis of the history of relations that lead up to the present situation (e.g., see Sammut et al., 2012), tracing the social and political ecology of an event, outlining the different intergroup relationships at play and understanding the relation to cultural narratives propagated in the media and on the street. Second, the researcher needs to access and analyze events as they change according to different contingencies and analyze how things emerge through time. In this way, we aim to understand the constraints and enablers of developing in a given direction. Third, researchers need to reflect on their own positionality and role in knowledge cocreation (Cornish, 2020a; Pedersen & Zittoun, 2022). Case studies are heterogeneous in their conceptualization and application. However, they share a focus on using ecologically valid qualitative techniques, including observations, participation, and forms of interview to explain, examine, or describe how people engage in their sociocultural world—in relation to a specific phenomenon—over time (e.g., Festinger et al., 1950; Jodelet, 1991; Sherif et al., 1961).

For example, one case study in Ireland, which included in-depth urban ethnographic research, semi-structured interviews, and triangulation of survey and media analyses, informed the understanding of how demonstrators in Ireland imagined an increasingly unfair economic future, when it became clear that economic recovery following drastic recession would benefit only a small minority. Multimethod qualitative studies, conducted over several years, revealed how these protesters feared the future privatization of water services in Ireland and how this commodification of what was seen as a public good—paid for through general taxation—would continue to increase economic inequalities in what was represented as an already unequal society (Power, 2017, 2018a, 2018b). Standard survey questions alone could not holistically comprehend the feared, and desired, futures imagined by the protesters because what was being imagined was novel within this context (Power, 2015). Instead, an inductive qualitative method was needed to capture processes of world-making. This occurred through demonstrators imagining a more unfair and unequal future, which looped back to the present to both justify and galvanize protest to mitigate this more dystopian future. This researcher also reflected on his own positionality with regard to knowledge creation and dissemination. And these ethical reflections informed widespread dissemination of this co-constructed knowledge (Power & Nussbaum, 2014, 2016) One consequence was to maximize the potential for world-making by presenting the research findings to a broad, international public who may or may not have reacted dialogically to these research findings to inform the course of the protest movement.

#### Surveys

Beyond creating knowledge to examine and understand world-making in relation to particular phenomena, ethnographic and qualitative case studies can be used to generate survey items that can quantify the responses of a broader range of respondents and their conceptualizations of world-making (e.g., Moscovici, 2008). Surveys can profitably address a range of world-making questions, including to what extent do people strive to make a better world (Basso & Krpan, 2022)? To what extent do people imagine the same collective future? What is shared, refuted, or debated, and what is misunderstood about our collective future orientation?

One way to examine imagination is to consider the moral foundations underlying visions for the future and their consequences for how people act in the present. One way to determine splintered visions for a moral future—held by different groups who occupy different positions—is to survey people about their attitudes, opinions, and perspectives on the future trajectory of their society. This method is common in political polling, for example, showing trends of political polarization and mutual radicalization between liberals and conservatives in the United States (Haidt, 2012; Moghaddam, 2018). Longitudinal surveys illustrate how people’s attitudes, opinions, and perspectives in the present reveal, and subsequently impact, the social and cultural worlds in which they live. For example, surveys have uncovered differences between and within countries in terms of people’s self-reporting of whether they would be vaccinated against Covid-19 (Lazarus et al., 2021). National and international media coverage of such survey results can validate the opinions of individuals and reflect those attitudes back to others en masse. People living in Denmark, for instance—in contrast to other EU countries, like France—have reported high levels of support for receiving a vaccine against the Covid-19 virus. Support for vaccination is reflected back via mainstream and social media and reinforces attitudes or offers a representation of a societal issue that needs to be remedied. In this sense, social scientific research is an integral part of a world-making dialogue between people, their governments, and potential vaccination.

#### Naturally Occurring Textual Data

Surveys are powerful in their standardization and precision, but they have two weaknesses from the standpoint of social psychology of world-making. First, they can only document the emergence of expected phenomena because the questions being asked are set by the researcher. Second, once a new phenomenon has emerged and is being tracked with a survey, it is not possible to go back in time and survey people before the phenomenon emerged. Both of these problems can be partially addressed by recent advances in natural language processing of naturally occurring textual data (Salganik, 2019). Algorithms, for example, can detect emerging topics in real time (e.g., how social media detects “trending” issues) by comparing current chatter with previous chatter. Social psychologists can use similar techniques to monitor changes in common sense, looping of social psychological theory into common sense, and emerging imaginations of our collective future (e.g., Glăveanu et al., 2018; Neuman, 2014). Moreover, once a new phenomenon begins to unfold, the algorithms can be applied retrospectively on any available textual data to provide a longitudinal analysis of the phenomenon’s emergence (Jordan et al., 2019). Moreover, algorithms that run in real time to detect emerging topics, trends, flaming, and incivility are all instances of looping, in that, the algorithm changes the course of online dialogues. Through these methodologies, social psychologists not only document the world as it is changing but also, through these examinations of the changing world, offer theories and explanations that themselves can lead to world-making.

### Methods for a Social Psychology *for* World-Making

Social psychology *for* world-making focuses on the creation of knowledge, not for mere understanding or prediction but for emancipation (Habermas, 1968). The aim is thus to develop knowledge that (to some extent) liberates humans from cognitive, communicative, interactive, and collective constraints. Instead of being passively guided by these processes, emancipatory knowledge enables humans to intervene in these processes, effectively enabling them to guide themselves into the future. Therefore, the focus is less on finding ultimate truths and more on enabling people to do the things they want to do and be the people they want to be. This formulation indicates at least two methods: action research and intervention research.

First, action research works with communities to address their problems (e.g., Cornish et al., in press; Engeström & Sannino, 2016). The aim of this method is to work with communities to identify and analyze problems and contradictions in practice and then build knowledge and envision and experiment with alternative ways of organizing the practice to address these problems and contradictions. Often, researchers conduct multimethod research, which has high levels of ecological validity. That is, what is observed, events that are participated in, and who is interviewed are closely linked to the community, group, or phenomenon under investigation. Next, analyses and interpretations of these data are then presented in one form or another to members of that community in an effort to reflect back academic knowledge to this community to help change a problematic issue, strive for equity and social justice, or otherwise improve—by explanation or shaping policy—the lives of inhabitants in these communities. This method of action research directly engages with positionality and plurality through continual reflexivity by remaining close to research participants.

One powerful example of action research is social scientific work on suicide in a small community in the United States, using qualitative and quantitative methods (Mueller & Abrutyn, 2015, 2016) These authors aimed to understand how high levels of integration and regulation shape suicide in modern societies. By focusing on a singular area with a disproportionally high level of adolescent suicide, they described and examined how adolescents in this tightly interconnected community were highly regulated by a local culture prizing academic achievement. The interconnected nature of this cohesive town meant gossip about academic successes and failures spread quickly through the community, adding to the potential emotional turmoil for the teenagers caused by perceived failures. Analyses and interpretations of the researchers’ observations and interviews were presented to people in the community who were bereaved or had indirectly experienced bereavement due to adolescent suicide in various meaningful forums. Moreover, community members were interviewed multiple times to enable them to voice their experiences and, importantly, their reactions to the initial analyses. This methodological choice was made both to enhance holistic knowledge of the phenomenon and to ensure that the thoughts and feelings of community members were adequately documented. In this way, through an ongoing relationship between the researchers and the researched, an effort was made to develop insights “for world-making,” that is, to change community dynamics to reduce and prevent further adolescent suicides.

Second, the focus on world-making resonates with intervention research, which involves explicit attempts at world-making. Intervention research often adopts a variety of quantitative methods, including randomized controlled trials, field experiments, and lab experiments. The explicit motivation for these types of studies is for world-making in a direction deemed congruent with social progress. There are examples across the social sciences, including behavioral scientific approaches to manipulating economic resources to promote prosocial behaviors (List & Gneezy, 2014; Thaler & Sunstein, 2009), the establishment of new social norms to promote peace in post-conflict zones (Paluck, 2009; Veale et al., 2013; Worthen et al., 2019), and the generation of improved learning conditions for children in the classroom (Velez et al., 2020).

From the standpoint of social psychology for world-making, intervention research is not secondary to fundamental research. Rather, it is the culmination of fundamental research. The most rigorous test of a theory is not whether it works in the laboratory, but whether it feeds forward through effective interventions into the future that people want (James, 1907). Firmly anchoring the validity of social psychological insights in applied consequences will help protect social psychology from trivial research questions and spurious findings.

Purposefully intervening in world-making has consequences not only for the world in which people live but also for psychological science. On the latter point, given that the knowledge created by social psychological research—especially research that is focused on understanding and then changing our socio-political, economic, or even legal realities—it is not surprising that the knock-on effects of our knowledge change the subjective realities in which we live. A failure to replicate the infamous Stanford Prison Experiment (Zimbardo, 2011), for example, or Milgram’s obedience studies (Milgram, 1974) might be due to the effects of these major findings altering people’s conceptualizations of power, context, and authority and leading people—and societies more generally—to question power and authority in different contexts to a greater degree than previous generations (Gibson, 2013; Haslam et al., 2019; Reicher & Haslam, 2006; Reicher et al., 2014). From this perspective, a failure to replicate can be understood as a victim of world-making; the sociocultural world moves on and a previous finding becomes history, with the new world becoming, in part, a response to that previous finding. In the final section, we conclude with a discussion of the implications, challenges, and possibilities of conceptualizing social psychology as a future-oriented discipline.

## Implications, Challenges, and Conclusions of a Social Psychology of and for World-Making

We have conceptualized social psychology as a discipline of and for world-making, discussing the ontological premise of such an advancement. The catalyst for this ontological re-orientation is based on two crises in contemporary social psychology: the replication crisis and the WEIRD problem. Both issues stem from having a static rather than a process ontology as their basis. Moreover, they also stem from publication pressures, access to participants, and inadequate sampling and statistical procedures, which are manifestations of the underlying issues. This essay does not attempt to solve these problems directly. Instead, we use them as a starting point to reimagine social psychology, with a processual, dialogical ontology. Our proposal is to start considering the social world as consisting of processes and phenomena that emerge and develop over time. This stands in contrast to dominant ontological commitments in social psychology and its typical constraints on generalizability. The mainstream approach tends to view the basic ontology of the social world as comprising static “things.” As such, social psychologists generally operate under an ontological assumption that experiments can capture all important and generalizable aspects of human life—like a photographer taking a picture of some reality. But, we argue the “social” in social psychology designates processes, relationships, and interactions and, as such, cannot be fully captured with an ontology of things.

Our proposal to have a future-oriented process ontology for social psychology has implications for the epistemological creation of social psychological knowledge. In contrast to an epistemology based on capturing thoughts, feelings, and behaviors in the present, we argue for the importance of showing how social psychological knowledge can be for, and of, future world-making. Our proposal—advancing recent articulations of psychology as a historical science—is to suggest it is a future-oriented science. This has methodological consequences. We document methodological frameworks both of and for world-making. We consider concrete contemporary research examples to illustrate our proposals. These methodological orientations overcome tensions between qualitative and quantitative methods and illustrate the potential utility of employing both in conjunction for world-making. Moreover, implicit in our methodological overview are ethical principles that overcome the standard separation between researcher and participants that is largely associated with a static ontological basis and most typically associated with research ethics in social psychology. Instead, we advocate for an ethical standpoint whereby the embeddedness of researchers—their socioeconomic positionings, interpretive biases, relative power, and privileges based on race, gender, and ethnicity—are critical, openly reflected upon, and acknowledged in world-making.

Given our argument for social psychology of and for world-making, one might ask what difference does all this theory make for research? How do these ideas about ontology, epistemology, ethics, and methods feed forward into new, interesting, and empowering research? Given the open-ended nature of world-making, our approach is not to prescribe theory, practices, or even research directions. Rather, we focus on heuristics and tricks of the trade (Becker, 2008) that can prompt social psychology of and for world-making. Specifically, we crystallize a series of “sensitizing questions” (Gillespie & Cornish, 2014). These are questions that researchers can ask themselves to reflexively interrogate their research practice and stimulate research of and for world-making. Table 1 enumerates key sensitizing questions at ontological, epistemological, ethical, and methodological levels.

**Table 1.** Guiding Questions for a Social Psychology of and for World-Making.

Level	Key question	Sub-questions
Ontology	Does the research foreground ‘things’ or ‘processes’?	Are the phenomena assumed to vary over time?
		What could cause changes over time?
		To what extent are the findings likely to be historical?
		If findings could vary, what would the consequences be?
Epistemology	What is the research basis for these findings?	Are phenomena naturally occurring or laboratory-based?
		What are the range of methods used?
		What are the consequences of the findings beyond research?
		How are the results shared with non-researchers?
Ethics	Who might be impacted by this knowledge?	Do the findings enable people to create own futures?
		Or do findings enable people to impose futures on others?
		Were the people impacted by knowledge involved?
		How will impacts be monitored and revised accordingly?
Methodology	Is the finding evident in both quantitative and qualitative data?	Do statistical associations manifest in concrete cases?
		Do the findings address participant concerns?
		Do the findings empower participants?
		Are the findings effective in real-world contexts?

At an ontological level, the key question is: Does the research foreground “things” or “processes”? For example, are the variables or observed phenomena assumed to vary over time? More specifically, what could make them change over time? How are these changes mutually related? And to what extent are the findings likely to be historical? What impacts might they have on the world? And if the findings could vary, then in which directions and with what consequences for people?

At an epistemological level, the key question is always: What is the basis for these findings? More specifically, we would encourage researchers to reflect on the breadth of the research base: Has the phenomenon been observed in naturally occurring human behavior or only in laboratory contexts? What range of methods have been used? Given that truth, in an everyday sense, is established through practical consequences, what consequence has the finding had for life outside the laboratory or beyond the specific fieldwork it was built in? How are the results shared with non-researchers, and what does the research enable them to do?

On an ethical level, the questions include: Who might be impacted by this knowledge? Do the findings enable people and groups to act upon themselves (e.g., create their own futures), or do they enable them to act upon others (e.g., impose their desired futures on others)? Given that it is impossible to know the unintended consequences of research findings, often the best way forward is inclusivity: Were the people who will be impacted by the knowledge involved in the creation of the knowledge? And what procedures are in place to monitor the impacts and revise the knowledge as necessary?

Finally, in terms of methods, the sensitizing questions focus on reducing the degrees of freedom. Responses to the replication crisis have proposed reducing degrees of freedom by clearly separating exploratory and confirmatory analyses, preregistration, and open data (Zwaan et al., 2018). In addition to these, we encourage researchers to ask: Is the finding evident in both quantitative and qualitative data? Do the statistical associations observed between columns of data points manifest in concrete cases? And do the findings speak to the participants, for example, address their concerns, solve their problems, or otherwise empower them?

As world-making social psychologists, we can explicitly inform, plan, and implement a specific change based on research contributing to organizational consultancy projects, law formation, or policy development. We can also account for a social change that is happening or that has taken place, and therefore, we can shape its possible consequences. We can also be involved in world-making in indirect ways, including diffusing social psychological knowledge through education and public outreach via the media or developing critical theories that envisage alternative forms of social life. Through such means, we participate in the collective work of world-imagining and world-making within and beyond the academy. There is a potential contradiction between advocating for well-intended interventions and arguing that the future consequences are unknowable. This is the tension at the heart of social psychology of and for world-making: We are not fully in control of the world-making process, yet we must take responsibility for it. First, this tension is familiar to all science, namely, creating knowledge based on past experience that is inevitably imperfect when applied to an unknown future. This does not imply giving up; rather, it implies continual refinement and revision. Second, this tension is particularly acute in the social sciences, where there is a long history of unintended consequences (Merton, 1936). To address this, we advocate for the broader participation of multiple voices in this process of reflection and refinement. Specifically, inviting research participants and anyone potentially impacted by the findings to participate in the critical reflection on the research will increase the chances of detecting unintended consequences early (e.g., unintended consequences for a minority group).

Conceptualizing social psychology of and for world-making leads not only to a generative discipline but also, as such, to the worlds in which we live. Extending the logic, inefficient or unsound research practices, and cultures (e.g., “publish or perish,” prioritizing surprising or counterintuitive findings, the power of funding sources to determine what knowledge is created) in social psychology can have negative societal consequences. Not only do unethical psychological research practices, such as p-hacking (selectively reporting data and analyses), HARKing (hypothesizing after the results are known; see Nelson et al., 2018), or oversampling WEIRD participants, lead to bad science, but also insights gleaned from these practices might be pushed through to inform policy and common sense. As such, it is not only “bad science” but also “bad world-making.”

However, in the surveys and intervention research we propose, people could still p-hack. In qualitative research, people might be tempted to twist things. The problem remains. One way that there could be a connection between methods and lived realities is if more historical data are used because these are public. One of the problems of the “replication crisis” is that data are replicated in a lab. But, if one takes a full turn to naturally occurring data, then all data are what they are (i.e., historical) and replication ceases to be an issue. The issue becomes why things have changed (i.e., the historicity is the phenomenon to explain, not the problem).

A realization that social psychology is not just a critical-historical science (Gergen, 1973; Sullivan, 2020) but a generative discipline that either implicitly or explicitly imagines and creates our socio-cultural worlds has implications for the types and forms of research projects, the funding bodies that support them, the types of theoretical and empirical knowledge produced within social psychology, and the impact the discipline will have.

World-making research should start by observing how people think, feel, and act within their sociocultural and historical contexts; what are the problems of living? How can lives be enriched? And researchers should reflect on how thoughts, feelings, and actions occur in relation to others, and how these interactions are acts of imagination and capable of world-making. Ethnographic methods, including observation, participation, and informal interview provide a useful starting point for documenting, describing, and examining these dynamic acts of world-making (e.g., Power & Velez, 2021). Accompanying these methodological practices is an implication that researchers are ethically responsible for knowledge creation and therefore world-making through their investigations and projects. By contributing these ontological, epistemological, ethical, and methodological insights and associated sensitizing questions, we aim to empower social psychologists not only to study people’s world-making but also to be part of making a more just, sustainable, and equitable world.

## Notes

1. We define social psychology throughout this article in its broadest sense: the study of individuals and context.
